# Crystal structure studies and Hirshfeld surface analysis of 4-(di­methyl­aza­nium­yl)-2-hy­droxy­anilinium dichloride monohydrate at 90 K

**DOI:** 10.1107/S2056989023007223

**Published:** 2023-08-23

**Authors:** Haleyur G. Anil Kumar, Thaluru M. Mohan Kumar, Thayamma R. Divakara, Doreswamy Geetha, Hemmige S. Yathirajan, Sean Parkin

**Affiliations:** a Department of Science and Humanities, PES University, BSK III Stage, Bengaluru-560 085, India; b Department of Chemistry, Amrita School of Engineering, Amrita Vishwa Vidyapeetham, Bengaluru-560 035, India; c T. John Institute of Technology, Bengaluru-560 083, India; d Department of Studies in Chemistry, University of Mysore, Manasagangotri, Mysuru-570 006, India; e Department of Chemistry, University of Kentucky, Lexington, KY, 40506-0055, USA; Katholieke Universiteit Leuven, Belgium

**Keywords:** crystal structure, aniline derivative, anilinium salt, hydrogen bonding

## Abstract

The crystal structure and a Hirshfeld surface analysis of the substituted anilinium salt 4-(di­methyl­aza­nium­yl)-2-hy­droxy­anilinium dichloride monohydrate, C_8_H_14_N_2_O^+^·2Cl^−^·H_2_O, at low temperature (90 K) are presented. The organic cation is essentially planar: the r.m.s. deviation of its non-hydrogen atoms (aside from the two methyl groups) is 0.0045 Å. The methyl carbons are 1.3125 (12) Å and 1.1278 (12) Å either side of the mean plane. The crystal packing involves extensive hydrogen bonding of types O—H⋯Cl, N—H⋯Cl, N—H⋯O_W_, and O_W_—H_W_⋯Cl (where W = water), which arrange into chains of *R* ^2^_4_(12) motifs that combine to form corrugated layers parallel to (10  ). Atom–atom contacts for the cation primarily involve hydrogen, leading to the most abundant coverage percentages being 51.3% (H⋯H), 23.0% (H⋯Cl), 12.9% (H⋯O), and 9.7% (C⋯H).

## Abstract 2

The low-temperature (90 K) crystal structure of 4-(di­methyl­aza­nium­yl)-2-hy­droxy­anilinium dichloride monohydrate is presented along with a Hirshfeld surface analysis of the organic cation.

## 1. Chemical context

Aniline is an important industrial feedstock chemical, broadly utilized throughout the chemical industry. For example, as a precursor to indigo, it is of paramount importance in the manufacture of dyes. Indeed, the modern synthetic dyestuffs industry traces its origin to mauveine, a product of William Henry Perkin’s attempts to synthesize quinine by oxidation of aniline (see *e.g.* Perkin, 1896 ▸). Aniline and its derivatives find extensive use in the rubber industry for processing materials used in products such as car tires, balloons, and gloves. In addition, aniline plays a crucial role in the production of numerous pharmaceutical drugs, including such well-known medications as paracetamol (*aka*, acetamino­phen/Tylenol) and the fenamate family of NSAIDs (anthranilic acid deriv­atives). Within this context, a concise review of aniline and its derivatives was presented by Anjalin *et al.* (2020 ▸). The hydrogen-bonding behavior of aniline derivatives has been investigated using FT–IR spectroscopy by Meng-Xia & Yuan (2002 ▸). The application of anilinium salts in polymer networks, resulting in materials with superior mechanical stability and mild thermally induced dynamic properties was reported by Chakma *et al.* (2019 ▸).

Given the industrial and pharmaceutical significance of anilinium salts, this paper presents the crystal structure and Hirshfeld-surface analysis of 4-(di­methyl­aza­nium­yl)-2-hy­droxy­anilinium dichloride monohydrate [C_8_H_14_N_2_O]^+^2Cl^−^·H_2_O (**I**), at 90 K. 
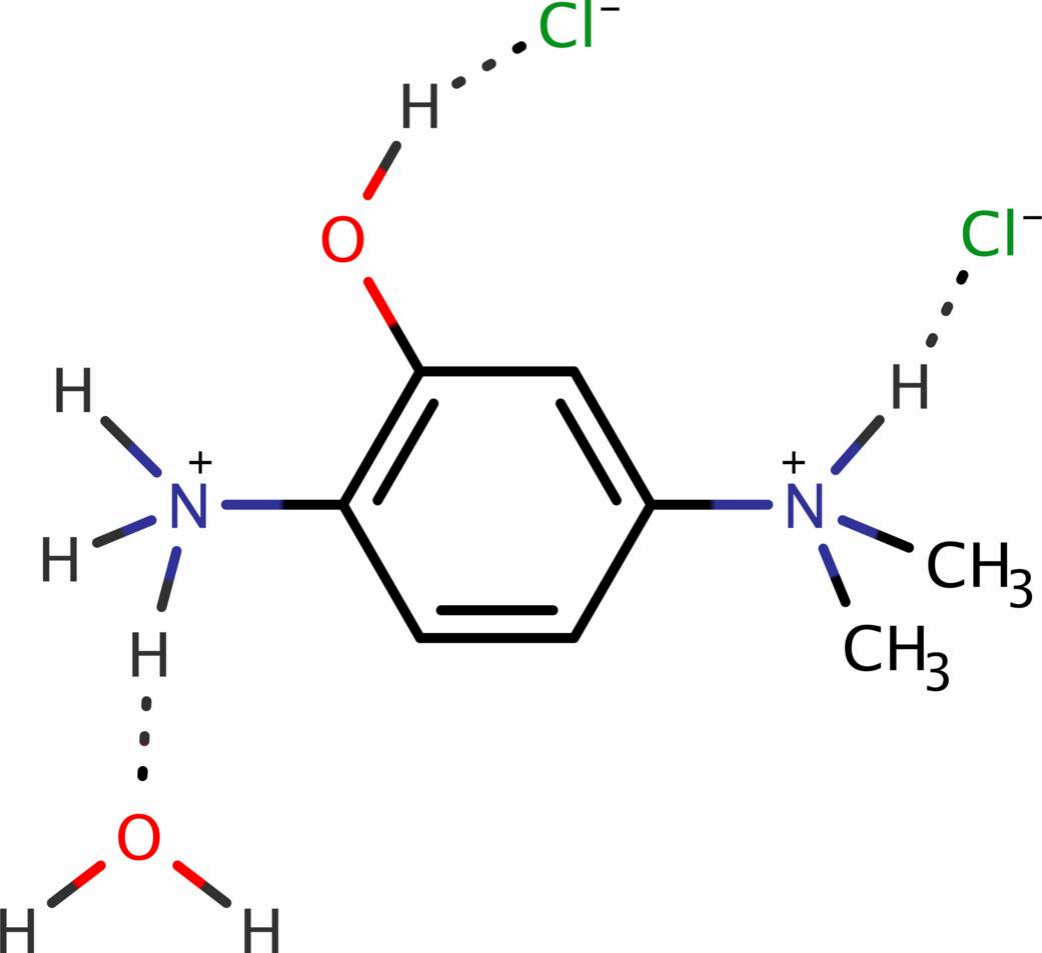


## 2. Structural commentary

**Figure 1:**
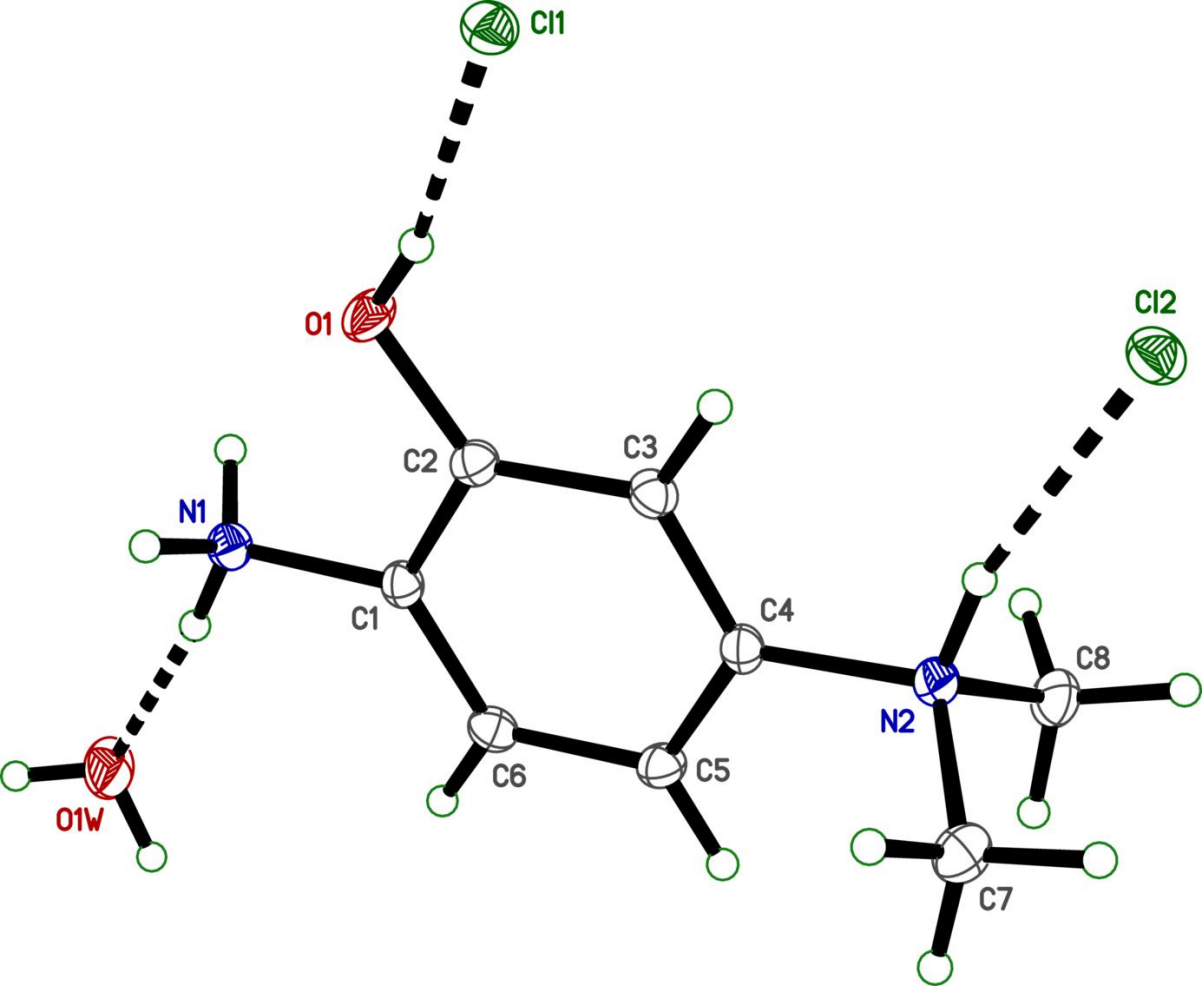
An ellipsoid plot (50% probability) of **I**. Hydrogen atoms are shown as small circles. Hydrogen bonds are drawn as dashed lines.

The asymmetric unit of **I** (see Fig. 1 ▸) consists of a single 4-(di­methyl­aza­nium­yl)-2-hy­droxy­anilinium dication, two chloride anions and a water of crystallization. The cation is largely planar. Aside from the two methyl groups, the r.m.s. deviation from the mean plane passing through the ring carbons, two nitro­gens and phenolic oxygen atom is 0.0045 Å, with the largest deviation being only 0.0096 (7) Å, for C5. The two methyl carbons lie 1.3125 (12) Å and 1.1278 (12) Å (for C7 and C8 respectively) either side of this mean plane. The water oxygen (O1*W*), at 0.1059 (14) Å, is also almost coplanar with the cation, while the chloride anions deviate by 0.4827 (12) Å (Cl1) and 0.4443 (12) Å (Cl2) to either side. The only inter­nal degree of freedom involves rotation of the di­methyl­aminium group about the C4—N2 bond, leading to torsion angles C3—C4—N2—C7 = 108.41 (9)°, C3—C4—N2—C8 = −125.32 (9)° and C3—C4—N2—H2*N* = −8.3 (8)°. There are no unusual bond lengths or angles in the structure.

## 3. Supra­molecular features

**Figure 2:**
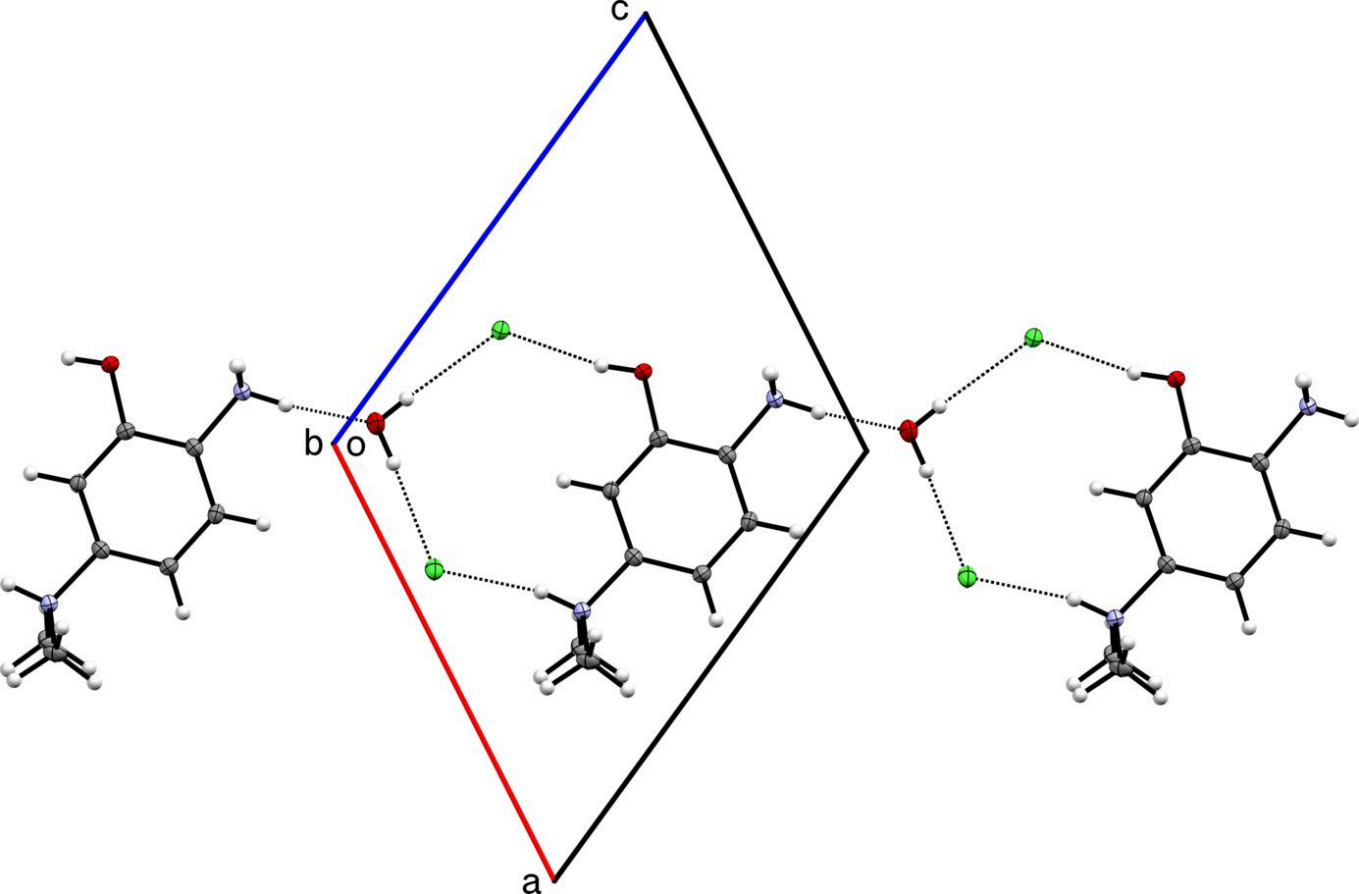
A partial packing plot of **I** showing 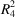 (12) hydrogen-bonded (dotted lines) ring motifs that link to form chains that propagate parallel to [101].

**Figure 3:**
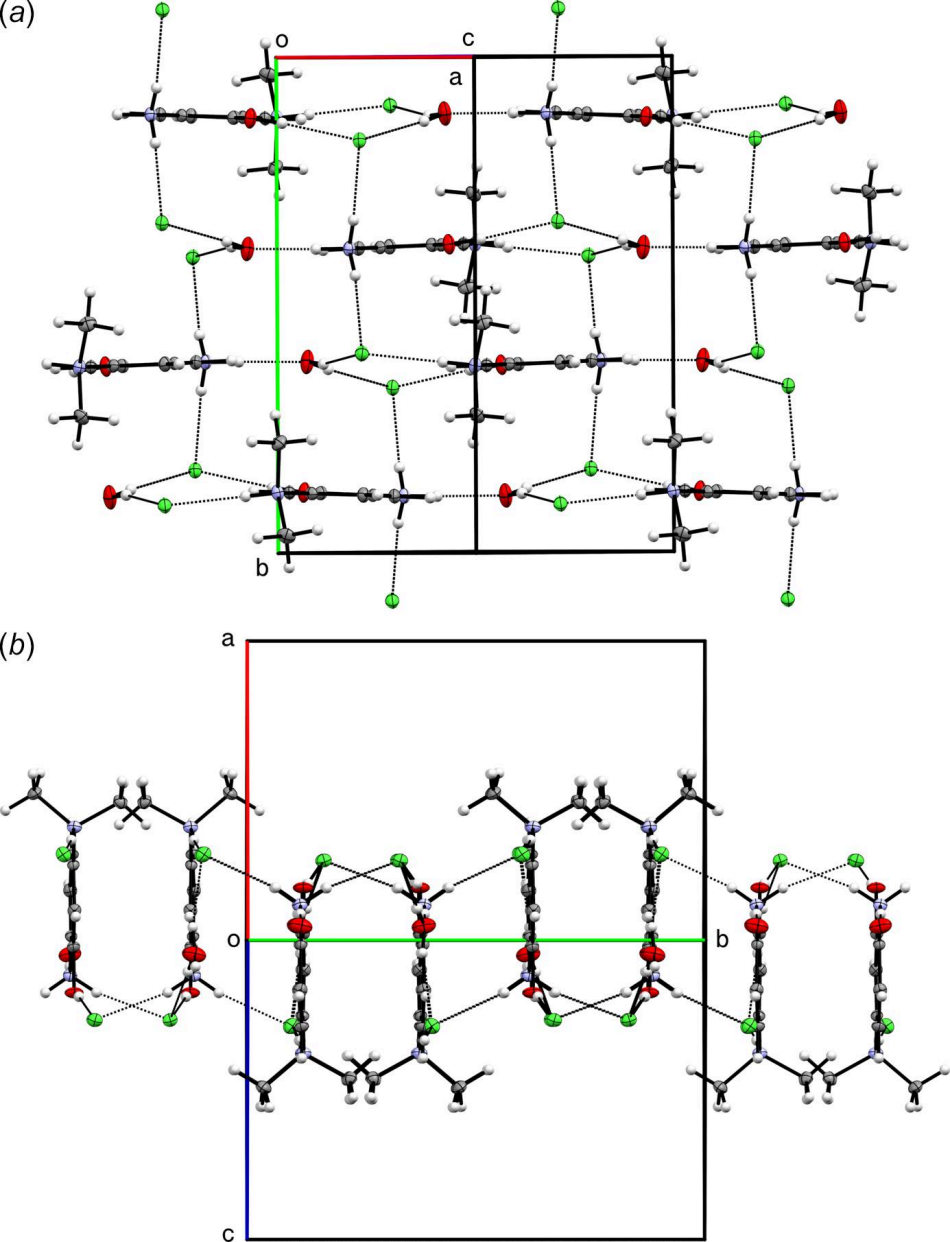
Partial packing plots of **I** showing: (*a*) hydrogen-bonded (dotted lines) layers that extend parallel to (10 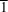 ) and (*b*) the same layers viewed side-on to highlight the corrugation.

Hydrogen-bonding inter­actions lead to the dominant structural features within the crystal packing of **I**, as qu­anti­fied in Table 1 ▸. Each organic cation engages in O1—H1*O*⋯Cl1 [*d_D–A_* = 2.9873 (8) Å] and N2—H2*N*⋯Cl2 [*d_D–A_* = 3.0467 (9) Å] hydrogen bonds with the chloride anions, which in turn act as acceptors for O1*W*—H1*W*1⋯Cl1^vi^ [*d_D–A_* = 3.1493 (9) Å] and O1*W*—H2*W*1⋯Cl2^vi^ [*d_D–A_* = 3.1036 (9) Å] hydrogen bonds with the water mol­ecule (symmetry codes as per Table 1 ▸). These inter­actions result in 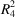 (12) motifs that link *via* N1—H3*N*1⋯O1*W* [*d_D–A_* = 2.7093 (12) Å] hydrogen bonds, forming chains that extend parallel to [101] (Fig. 2 ▸). These chains are connected by N1—H1*N*1⋯Cl1^ii^ [*d_D–A_* = 3.1364 (9) Å] and N1—H1*N*1⋯Cl2^i^ [*d_D–A_* = 3.1299 (9) Å] hydrogen bonds, forming corrugated layers parallel to (10 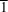 ) (Fig. 3 ▸).

**Figure 4:**
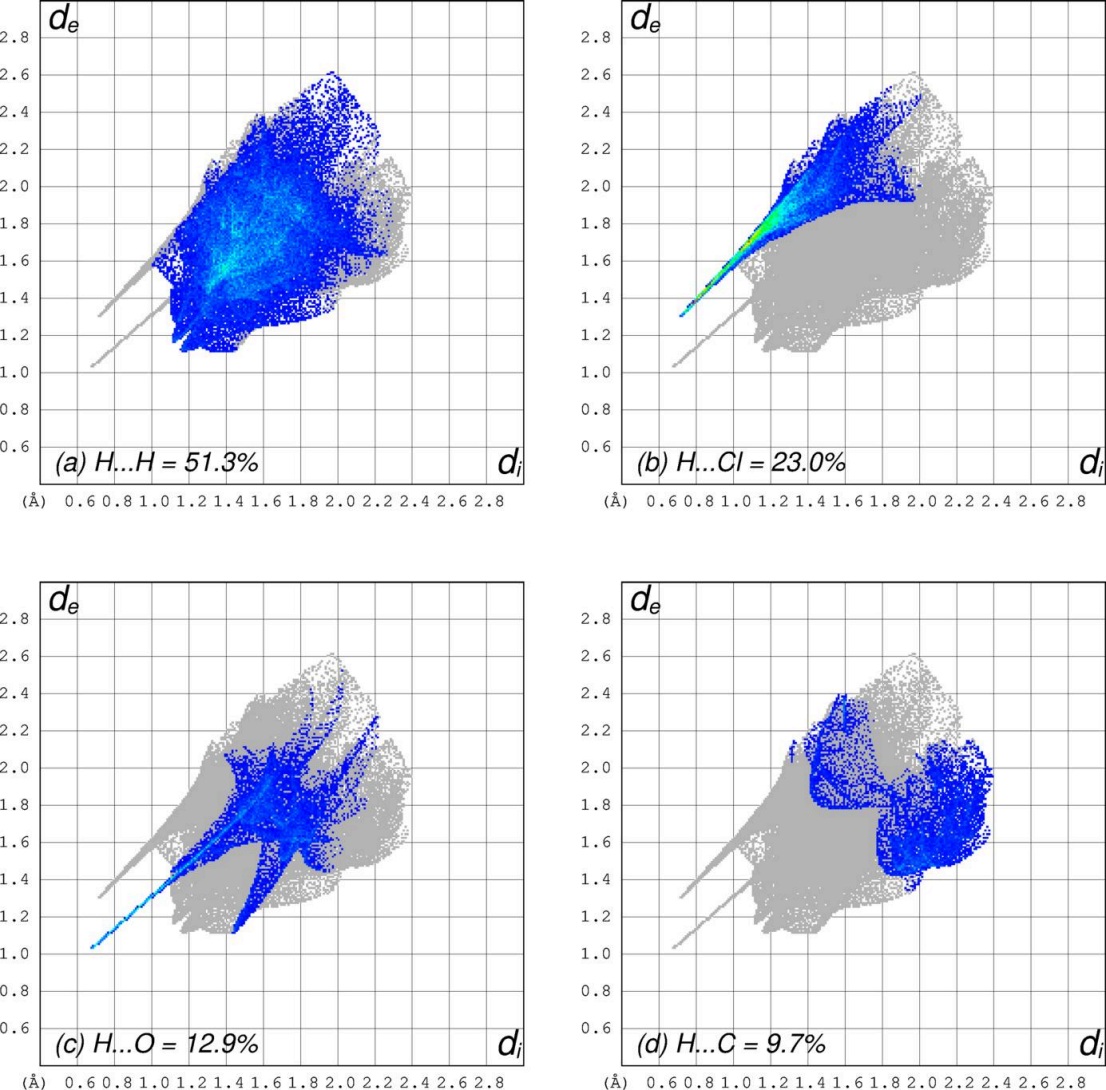
Two-dimensional fingerprint plots from a Hirshfeld-surface analysis of the cations in **I** showing: (*a*) H⋯H contacts (51.3%); (*b*) H⋯Cl/Cl⋯H (23.0%); (*c*) H⋯O/O⋯H (12.9%); (*d*) H⋯C/C⋯H (9.7%).

Two-dimensional fingerprint plots (Fig. 4 ▸) derived from a Hirshfeld surface analysis mapped over *d_norm_* for the cation in **I** were obtained using *CrystalExplorer* (Spackman *et al.*, 2021 ▸). These show that atom–atom contacts for the cation are dominated by hydrogen, either to other H atoms (51.3%) or to Cl (23.0%), O (12.9%), or C (9.7%), all other types giving negligible coverage.

## 4. Database survey

A search of the Cambridge Structural Database (CSD, v5.43 with all updates to November 2022; Groom *et al.*, 2016 ▸) for a mol­ecular fragment composed of a benzene ring with any N-bound group at the 1- and 4-positions and an O-bound group at the 2-position yielded 471 matches. With the O-bound group defined as hydroxyl there were 62 hits. The further restriction of having two C-bound groups attached to the 4-N nitro­gen returned 15 entries (13 unique), but with the C-bound groups both specified as methyl there were no matches. Of the 13 unique structures only one, XAVKAJ [(C_30_H_32_N_6_O_2_)^4+^·4Cl^−^·4H_2_O; Stylianou *et al.*, 2017 ▸] is a salt or a hydrate, but it has little else in common with **I**. Two other anilinium salts not returned in the above search but that share similar features to **I** are POMXUL (Smirani & Rzaigui, 2009 ▸), or 2,5-di­methyl­anilinium chloride monohydrate (C_8_H_12_N^+^·Cl^−^·H_2_O) and PAXXIX (Devi *et al.*, 2012 ▸), which is 4-[(*E*)-(hy­droxy­imino)­meth­yl]-*N*,*N*-dimethyl anilinium chloride (C_9_H_13_N_2_O^+^·Cl^−^).

## 5. Synthesis and crystallization

The sample of **I** was obtained as a gift from Honeychem Pharma, Bengaluru, India. Crystals suitable for X-ray structure determination were obtained from a solution in ethanol by slow evaporation.

## 6. Refinement

Crystal data, data collection and structure refinement details are summarized in Table 2 ▸. All hydrogen atoms were present in difference-Fourier maps. Carbon-bound hydrogens were subsequently included in the refinement using riding models, with constrained distances of 0.95 Å (*R*_2_CH) and 0.98 Å (*R*CH_3_) and *U*_iso_(H) parameters set to either 1.2*U*_eq_ (*R*_2_CH) or 1.5*U*_eq_ (*R*CH_3_) of the attached carbon. Nitro­gen and oxygen-bound hydrogens were fully refined (*x*, *y*, *z*, and *U*_iso_).

## Supplementary Material

Crystal structure: contains datablock(s) I, global. DOI: 10.1107/S2056989023007223/vm2289sup1.cif

Structure factors: contains datablock(s) I. DOI: 10.1107/S2056989023007223/vm2289Isup2.hkl

CCDC reference: 2289098

Additional supporting information:  crystallographic information; 3D view; checkCIF report

## Figures and Tables

**Table 1:** Hydrogen-bond geometry (Å, °)

*D*—H⋯*A*	*D*—H	H⋯*A*	*D*⋯*A*	*D*—H⋯*A*
N1—H2*N*1⋯Cl2^i^	0.873 (14)	2.270 (14)	3.1299 (9)	168.5 (12)
N1—H1*N*1⋯Cl1^ii^	0.895 (14)	2.257 (14)	3.1364 (9)	167.4 (12)
N1—H3*N*1⋯O1*W*	0.893 (17)	1.819 (17)	2.7093 (12)	174.4 (14)
O1—H1*O*⋯Cl1	0.843 (18)	2.156 (18)	2.9873 (8)	168.9 (15)
N2—H2*N*⋯Cl2	0.903 (15)	2.161 (15)	3.0467 (9)	166.7 (12)
C5—H5⋯Cl1^iii^	0.95	2.98	3.8846 (10)	160
C7—H7*A*⋯Cl1^iv^	0.98	2.78	3.6641 (10)	151
C7—H7*B*⋯Cl1^iii^	0.98	2.79	3.7079 (11)	156
C8—H8*A*⋯Cl2^v^	0.98	2.82	3.6774 (10)	147
C8—H8*B*⋯O1^i^	0.98	2.64	3.4610 (13)	142
C8—H8*C*⋯Cl1^iii^	0.98	2.87	3.7809 (11)	156
O1*W*—H2*W*1⋯Cl2^vi^	0.857 (18)	2.259 (18)	3.1036 (9)	168.7 (13)
O1*W*—H1*W*1⋯Cl1^vi^	0.802 (19)	2.348 (19)	3.1493 (9)	176.4 (16)

Symmetry codes: (i) 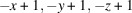 ; (ii) 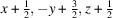 ; (iii) 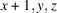 ; (iv) 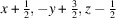 ; (v) 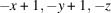 ; (vi) 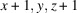 .

**Table 2:** Experimental details

Crystal data	
Chemical formula	C_8_H_14_N_2_O^2+^·2Cl^−^·H_2_O
*M*_r_	243.13
Crystal system, space group	Monoclinic, *P*2_1_/*n*
Temperature (K)	90
*a*, *b*, *c* (Å)	9.6493 (7), 13.0873 (8), 10.4634 (7)
β (°)	117.188 (2)
*V* (Å^3^)	1175.36 (14)
*Z*	4
Radiation type	Mo *K*α
μ (mm^−1^)	0.53
Crystal size (mm)	0.32 × 0.30 × 0.22

Data collection	
Diffractometer	Bruker D8 Venture dual source
Absorption correction	Multi-scan (*SADABS*; Krause *et al.*, 2015 ▸)
*T*_min_, *T*_max_	0.888, 0.971
No. of measured, independent and observed [*I* > 2σ(*I*)] reflections	36719, 2693, 2505
*R*_int_	0.032
(sin θ/λ)_max_ (Å^−1^)	0.650

Refinement	
*R*[*F* ^2^ > 2σ(*F* ^2^)], *wR*(*F* ^2^), *S*	0.021, 0.063, 1.13
No. of reflections	2693
No. of parameters	158
H-atom treatment	H atoms treated by a mixture of independent and constrained refinement
Δρ_max_, Δρ_min_ (e Å^−3^)	0.34, −0.22

Computer programs: *APEX3* (Bruker, 2016 ▸), *SHELXT* (Sheldrick, 2015*a* ▸), *SHELXL2019/2* (Sheldrick, 2015*b* ▸), *XP* in *SHELXTL* (Sheldrick, 2008 ▸), *SHELX* (Sheldrick, 2008 ▸) and *publCIF* (Westrip, 2010 ▸).

## supplementary crystallographic information

### Crystal data

**Table:**

C_8_H_14_N_2_O^2+^·2Cl^−^·H_2_O	*F*(000) = 512
*M**_r_* = 243.13	*D*_x_ = 1.374 Mg m^−^^3^
Monoclinic, *P*2_1_/*n*	Mo *K*α radiation, λ = 0.71073 Å
*a* = 9.6493 (7) Å	Cell parameters from 9459 reflections
*b* = 13.0873 (8) Å	θ = 2.4–27.5°
*c* = 10.4634 (7) Å	µ = 0.53 mm^−^^1^
β = 117.188 (2)°	*T* = 90 K
*V* = 1175.36 (14) Å^3^	Irregular block, colourless
*Z* = 4	0.32 × 0.30 × 0.22 mm

### Data collection

**Table:**

Bruker D8 Venture dual source diffractometer	2693 independent reflections
Radiation source: microsource	2505 reflections with *I* > 2σ(*I*)
Detector resolution: 7.41 pixels mm^-1^	*R*_int_ = 0.032
φ and ω scans	θ_max_ = 27.5°, θ_min_ = 2.4°
Absorption correction: multi-scan (*SADABS*; Krause *et al.*, 2015)	*h* = −12→12
*T*_min_ = 0.888, *T*_max_ = 0.971	*k* = −16→17
36719 measured reflections	*l* = −13→13

### Refinement

**Table:**

Refinement on *F*^2^	Secondary atom site location: difference Fourier map
Least-squares matrix: full	Hydrogen site location: mixed
*R*[*F*^2^ > 2σ(*F*^2^)] = 0.021	H atoms treated by a mixture of independent and constrained refinement
*wR*(*F*^2^) = 0.063	*w* = 1/[σ^2^(*F*_o_^2^) + (0.034*P*)^2^ + 0.2669*P*] where *P* = (*F*_o_^2^ + 2*F*_c_^2^)/3
*S* = 1.13	(Δ/σ)_max_ = 0.001
2693 reflections	Δρ_max_ = 0.34 e Å^−^^3^
158 parameters	Δρ_min_ = −0.22 e Å^−^^3^
0 restraints	Extinction correction: SHELXL-2019/2 (Sheldrick 2015b), Fc^*^=kFc[1+0.001xFc^2^λ^3^/sin(2θ)]^-1/4^
Primary atom site location: structure-invariant direct methods	Extinction coefficient: 0.008 (2)

### Special details

**Table:**

Experimental. The crystal was mounted using polyisobutene oil on the tip of a fine glass fibre, which was fastened in a copper mounting pin with electrical solder. It was placed directly into the cold gas stream of a liquid-nitrogen based cryostat (Hope, 1994; Parkin & Hope, 1998). Diffraction data were collected with the crystal at 90K, which is standard practice in this laboratory for the majority of flash-cooled crystals.
Geometry. All esds (except the esd in the dihedral angle between two l.s. planes) are estimated using the full covariance matrix. The cell esds are taken into account individually in the estimation of esds in distances, angles and torsion angles; correlations between esds in cell parameters are only used when they are defined by crystal symmetry. An approximate (isotropic) treatment of cell esds is used for estimating esds involving l.s. planes.
Refinement. Refinement progress was checked using *Platon* (Spek, 2020) and by an *R*-tensor (Parkin, 2000). The final model was further checked with the IUCr utility *checkCIF*.

### Fractional atomic coordinates and isotropic or equivalent isotropic displacement parameters (Å^2^)

**Table:**

	*x*	*y*	*z*	*U*_iso_*/*U*_eq_	
N1	0.75930 (11)	0.61398 (6)	0.87704 (9)	0.01338 (18)	
H1N1	0.7194 (15)	0.6712 (11)	0.8938 (14)	0.022 (3)*	
H2N1	0.7151 (15)	0.5598 (11)	0.8907 (13)	0.023 (3)*	
H3N1	0.858 (2)	0.6134 (10)	0.9459 (17)	0.028 (4)*	
C1	0.74643 (11)	0.61590 (6)	0.73284 (10)	0.01215 (19)	
O1	0.47817 (9)	0.62201 (5)	0.65346 (8)	0.01714 (17)	
H1O	0.392 (2)	0.6321 (11)	0.5805 (18)	0.037 (4)*	
Cl1	0.15713 (2)	0.66738 (2)	0.42367 (2)	0.01449 (9)	
C2	0.59693 (11)	0.62072 (7)	0.61890 (11)	0.0128 (2)	
N2	0.68764 (10)	0.62236 (6)	0.30958 (9)	0.01355 (18)	
H2N	0.5849 (17)	0.6154 (9)	0.2501 (15)	0.021 (3)*	
Cl2	0.35654 (3)	0.59763 (2)	0.06980 (2)	0.01665 (9)	
C3	0.57844 (11)	0.62244 (7)	0.47882 (11)	0.0132 (2)	
H3	0.477593	0.625003	0.399057	0.016*	
C4	0.71073 (12)	0.62031 (7)	0.45870 (11)	0.0123 (2)	
C5	0.85995 (11)	0.61711 (7)	0.57131 (11)	0.0137 (2)	
H5	0.948406	0.616984	0.554007	0.016*	
C6	0.87688 (11)	0.61406 (7)	0.71058 (11)	0.0136 (2)	
H6	0.977827	0.610731	0.790114	0.016*	
C7	0.73470 (12)	0.72278 (8)	0.27212 (11)	0.0172 (2)	
H7A	0.699595	0.725867	0.168286	0.026*	
H7B	0.848358	0.729470	0.323059	0.026*	
H7C	0.686888	0.778611	0.300525	0.026*	
C8	0.76558 (12)	0.53561 (8)	0.27443 (11)	0.0184 (2)	
H8A	0.725765	0.531196	0.170044	0.028*	
H8B	0.743641	0.471597	0.310319	0.028*	
H8C	0.878337	0.547275	0.319913	0.028*	
O1W	1.05118 (10)	0.61432 (6)	1.09862 (9)	0.0249 (2)	
H1W1	1.075 (2)	0.6295 (12)	1.180 (2)	0.041 (5)*	
H2W1	1.137 (2)	0.6187 (10)	1.0942 (18)	0.035 (4)*	

### Atomic displacement parameters (Å^2^)

**Table:**

	*U*^11^	*U*^22^	*U*^33^	*U*^12^	*U*^13^	*U*^23^
N1	0.0133 (4)	0.0154 (4)	0.0105 (4)	0.0000 (3)	0.0046 (4)	0.0005 (3)
C1	0.0142 (5)	0.0115 (4)	0.0107 (4)	−0.0004 (3)	0.0056 (4)	0.0000 (3)
O1	0.0104 (4)	0.0288 (4)	0.0127 (4)	0.0019 (3)	0.0057 (3)	0.0013 (3)
Cl1	0.01198 (13)	0.01708 (14)	0.01299 (13)	−0.00068 (8)	0.00449 (10)	0.00011 (8)
C2	0.0121 (5)	0.0132 (4)	0.0136 (5)	0.0002 (3)	0.0063 (4)	0.0000 (3)
N2	0.0119 (4)	0.0173 (4)	0.0112 (4)	−0.0011 (3)	0.0050 (3)	−0.0009 (3)
Cl2	0.01496 (14)	0.01780 (14)	0.01421 (14)	−0.00158 (8)	0.00409 (10)	0.00050 (8)
C3	0.0113 (4)	0.0146 (5)	0.0121 (5)	0.0002 (3)	0.0038 (4)	−0.0006 (3)
C4	0.0149 (5)	0.0116 (4)	0.0108 (4)	−0.0004 (3)	0.0061 (4)	−0.0003 (3)
C5	0.0113 (5)	0.0157 (5)	0.0145 (5)	−0.0002 (3)	0.0062 (4)	0.0003 (3)
C6	0.0108 (5)	0.0147 (5)	0.0128 (5)	−0.0002 (3)	0.0031 (4)	0.0002 (3)
C7	0.0190 (5)	0.0174 (5)	0.0157 (5)	0.0015 (4)	0.0084 (4)	0.0044 (4)
C8	0.0227 (5)	0.0180 (5)	0.0192 (5)	−0.0004 (4)	0.0135 (4)	−0.0039 (4)
O1W	0.0160 (4)	0.0415 (5)	0.0139 (4)	−0.0006 (3)	0.0039 (3)	−0.0056 (3)

### Geometric parameters (Å, º)

**Table:**

N1—C1	1.4565 (12)	C3—H3	0.9500
N1—H1N1	0.895 (14)	C4—C5	1.3831 (14)
N1—H2N1	0.873 (14)	C5—C6	1.3908 (14)
N1—H3N1	0.893 (17)	C5—H5	0.9500
C1—C6	1.3810 (14)	C6—H6	0.9500
C1—C2	1.3909 (14)	C7—H7A	0.9800
O1—C2	1.3502 (12)	C7—H7B	0.9800
O1—H1O	0.843 (18)	C7—H7C	0.9800
C2—C3	1.3934 (14)	C8—H8A	0.9800
N2—C4	1.4721 (12)	C8—H8B	0.9800
N2—C8	1.4976 (12)	C8—H8C	0.9800
N2—C7	1.4998 (12)	O1W—H1W1	0.802 (19)
N2—H2N	0.903 (15)	O1W—H2W1	0.857 (18)
C3—C4	1.3857 (14)		
			
C1—N1—H1N1	110.3 (8)	C5—C4—C3	122.95 (9)
C1—N1—H2N1	111.5 (8)	C5—C4—N2	119.86 (9)
H1N1—N1—H2N1	111.2 (13)	C3—C4—N2	117.19 (9)
C1—N1—H3N1	113 (1)	C4—C5—C6	118.14 (9)
H1N1—N1—H3N1	104.1 (12)	C4—C5—H5	120.9
H2N1—N1—H3N1	106.3 (12)	C6—C5—H5	120.9
C6—C1—C2	121.64 (9)	C1—C6—C5	119.76 (9)
C6—C1—N1	121.44 (9)	C1—C6—H6	120.1
C2—C1—N1	116.93 (9)	C5—C6—H6	120.1
C2—O1—H1O	111.4 (11)	N2—C7—H7A	109.5
O1—C2—C1	116.47 (9)	N2—C7—H7B	109.5
O1—C2—C3	124.41 (9)	H7A—C7—H7B	109.5
C1—C2—C3	119.11 (9)	N2—C7—H7C	109.5
C4—N2—C8	113.37 (8)	H7A—C7—H7C	109.5
C4—N2—C7	112.07 (7)	H7B—C7—H7C	109.5
C8—N2—C7	110.74 (8)	N2—C8—H8A	109.5
C4—N2—H2N	108.4 (9)	N2—C8—H8B	109.5
C8—N2—H2N	105.7 (8)	H8A—C8—H8B	109.5
C7—N2—H2N	106.1 (8)	N2—C8—H8C	109.5
C4—C3—C2	118.39 (9)	H8A—C8—H8C	109.5
C4—C3—H3	120.8	H8B—C8—H8C	109.5
C2—C3—H3	120.8	H1W1—O1W—H2W1	103.4 (16)
			
C6—C1—C2—O1	179.93 (8)	C7—N2—C4—C5	−71.16 (11)
N1—C1—C2—O1	−0.70 (12)	C8—N2—C4—C3	−125.32 (9)
C6—C1—C2—C3	0.81 (13)	C7—N2—C4—C3	108.41 (9)
N1—C1—C2—C3	−179.82 (8)	C3—C4—C5—C6	1.17 (13)
O1—C2—C3—C4	−179.70 (8)	N2—C4—C5—C6	−179.27 (8)
C1—C2—C3—C4	−0.65 (13)	C2—C1—C6—C5	0.03 (13)
C2—C3—C4—C5	−0.34 (13)	N1—C1—C6—C5	−179.31 (8)
C2—C3—C4—N2	−179.91 (8)	C4—C5—C6—C1	−0.99 (13)
C8—N2—C4—C5	55.10 (11)		

### Hydrogen-bond geometry (Å, º)

**Table:**

*D*—H···*A*	*D*—H	H···*A*	*D*···*A*	*D*—H···*A*
N1—H2*N*1···Cl2^i^	0.873 (14)	2.270 (14)	3.1299 (9)	168.5 (12)
N1—H1*N*1···Cl1^ii^	0.895 (14)	2.257 (14)	3.1364 (9)	167.4 (12)
N1—H3*N*1···O1*W*	0.893 (17)	1.819 (17)	2.7093 (12)	174.4 (14)
O1—H1*O*···Cl1	0.843 (18)	2.156 (18)	2.9873 (8)	168.9 (15)
N2—H2*N*···Cl2	0.903 (15)	2.161 (15)	3.0467 (9)	166.7 (12)
C5—H5···Cl1^iii^	0.95	2.98	3.8846 (10)	160
C7—H7*A*···Cl1^iv^	0.98	2.78	3.6641 (10)	151
C7—H7*B*···Cl1^iii^	0.98	2.79	3.7079 (11)	156
C8—H8*A*···Cl2^v^	0.98	2.82	3.6774 (10)	147
C8—H8*B*···O1^i^	0.98	2.64	3.4610 (13)	142
C8—H8*C*···Cl1^iii^	0.98	2.87	3.7809 (11)	156
O1*W*—H2*W*1···Cl2^vi^	0.857 (18)	2.259 (18)	3.1036 (9)	168.7 (13)
O1*W*—H1*W*1···Cl1^vi^	0.802 (19)	2.348 (19)	3.1493 (9)	176.4 (16)

Symmetry codes: (i) −*x*+1, −*y*+1, −*z*+1; (ii) *x*+1/2, −*y*+3/2, *z*+1/2; (iii) *x*+1, *y*, *z*; (iv) *x*+1/2, −*y*+3/2, *z*−1/2; (v) −*x*+1, −*y*+1, −*z*; (vi) *x*+1, *y*, *z*+1.
